# Sonication accelerated formation of Mg-Al-phosphate layered double hydroxide via sol-gel prepared mixed metal oxides

**DOI:** 10.1038/s41598-019-46910-5

**Published:** 2019-07-18

**Authors:** Denis Sokol, Daniel E. L. Vieira, Aleksej Zarkov, Mário G. S. Ferreira, Aldona Beganskiene, Vasili V. Rubanik, Aleksandr D. Shilin, Aivaras Kareiva, Andrei N. Salak

**Affiliations:** 1 Institute of Chemistry, Faculty of Chemistry and Geosciences, Vilnius University, Naugarduko 24, LT-03225 Vilnius, Lithuania; 2 Department of Materials and Ceramics Engineering and CICECO – Aveiro Institute of Materials, University of Aveiro, 3810-193 Aveiro, Portugal; 3 Institute of Technical Acoustics of National Academy of Sciences of Belarus, Lyudnikov Avenue, 13, 210009 Vitebsk, Belarus

**Keywords:** Two-dimensional materials, Nanoparticle synthesis, Two-dimensional materials, Nanoparticle synthesis

## Abstract

Single-phase magnesium-aluminium layered double hydroxide (LDH) intercalated with dihydrogen phosphate was successfully produced by hydration of nanopowder of the respective mixed metal oxide (MMO) obtained using sol-gel based method followed by a two-step anion exchange hydroxide-to-chloride and chloride-to-phosphate. The MMO with the metal cation ratio of Mg/Al = 2:1 was prepared using the aqueous sol-gel method. Processes of the parent Mg_2_Al-OH LDH formation and the successive anion-exchanges, ОН^−^ → Cl^−^ and Cl^−^ → H_2_PO_4_^−^, were considerably accelerated via the application of high-power (1.5 kW) ultrasound. The crystalline phases formed at all stages of the Mg_2_Al-H_2_PO_4_ LDH production were characterized using X-ray diffraction, scanning electron microscopy, scanning transmission electron microscopy, inductive coupled plasma optical emission spectroscopy, Fourier transform infrared spectroscopy, and thermogravimetric analysis. Based on the data of chemical analysis and the XRD data, the type of the intercalated phosphate anion was determined and the arrangement of this anion in the interlayer was modelled.

## Introduction

In layered double hydroxides (LDHs), the positively charged mixed metal cation hydroxide layer alternate with charge-compensating interlayer of anions A^*m*−^ coordinated by water molecules^1^. Although M^I^-M^III^ LDHs are known^2,3^, the great majority of layered hydroxides are of the M^II^-M^III^ type. The generic chemical formula of such LDHs can be represented as [M^II^_1-*x*_ M^III^_*x*_(OH)_2_]^*x*+^(A^*m*−^)_*x*/*m*_·*z*H_2_O^1^ (hereafter M^II^_*n*_M^III^-A, where *n* = (1 − *x*)/*x*). The M^II^ cation is usually magnesium or a 4^th^-period transition metal from iron to zinc, and M^III^ is, as a rule, Al, Ga, Fe, or Cr^3^. It has recently been demonstrated that some amount (an order of 10 mol%) of large trivalent cations, namely lanthanides^4–6^ and bismuth^7^ can substitute M^III^ in the LDH structure. The most used LDHs have the M^II^/M^III^ cations ratio between 2 and 3, although it can be in the range from 1 to about 5^1^. Different layer charge density conditioned by the cations ratio and the natural flexibility of the crystal structure allows formation of LDHs intercalated with a great variety of inorganic anions [ref.^3^ and the references therein], among them the most occurred are Cl^−^^8,9^, OH^−^^10^, CO_3_^2−^^11^, PO_4_^3−^^12–14^, and organic anions [ref.^3^ and the references therein^15^].

Layered double hydroxides have found various applications in many areas, such as catalysis^16^, drug delivery^17^, adsorption^18^, separation^19^, energy storage^20^, hydrogen and oxygen evolution reactions^21^, and corrosion protection^22^. Majority of the commercially produced LDHs are made by co-precipitation^23^, by hydrothermal synthesis^24^ or via the route that combines both these methods^25^. All these three mentioned techniques make possible a production of well-crystalized product with good reproducibility; however, they are rather time-consuming.

Recently, we have developed an environment-friendly method for the fabrication of LDHs with more than one type of the M^III^ cation in the hydroxide layer^5–7^. In this novel aqueous sol-gel processing route, LDHs are obtained as a result of the decomposition (calcination) of the precursor gels at 650^o^C followed by rehydration of the intermediate crystalline mixed metal oxide (MMO) powders in water. This method allows us not only to produce LDHs with different sets of cations, but also with the desired M^II^/M^III^ ratio. This feature is fundamental for control of the LDH anion capacity and exchange ability. The main drawback of the sol-gel based method is that the LDH formed as a result of rehydration of the respective MMO is intercalated with OH^−^ regardless of the presence of other anions (except for CO_3_^2−^) in the solution. There is a problem to substitute hydroxide and carbonate to another anions, including the functional ones, since OH^−^ and CO_3_^2−^ show strong affinity towards the double-metal hydroxide layer^26^. In 2004, Iyi *et al*. reported successful deintercalation of carbonate from the commercially available Mg_3_Al-CO_3_ hydrotalcite using the HCl/NaCl acid-salt solution^27^. One can assume that the same or similar method can be applied to substitute hydroxide for chloride in Mg_2_Al-OH LDH.

Several methods were applied to optimise the LDH preparation processes. It has been shown that sonication assists rehydration of hydrotalcite via a “shape-memory”-like reaction^28^. Besides, sonication was applied at the stage of synthesis of Mg_3_Al-CO_3_ to promote a formation of uniform LDH microparticles^29^. Ultrasound was also used in the LDH formation to assist synthesis^30^, anion-exchange reactions^31,32^ and in the LDH functionalization^33,34^. It should be stressed here that in all the aforementioned cases, the applied sonication power was rather moderate (an order of 100 W) as compared with that employed in this work (1.5 kW).

The structural data on phosphate-containing LDHs available from literature are rather controversial^12,35–37^. This appears to relate to a diversity of phosphate species in water-based solutions and to difficulties in the identification of the type and arrangement of those species in the interlayer. Besides, in some cases, grafting of phosphate anions in hydroxide layer is suggested^35^. Badreddine *et al*.^36^ reported the basal spacings (which are the distances between the adjacent hydroxide layers) of Zn_2_Al LDHs obtained as a result of chloride-to-phosphate anion exchange depending on pH of the exchange solution. However, the obtained basal spacing values were not correlated with the dimensions of the intercalated phosphate anions.

In this work, we have combined the aqueous sol-gel based method of production of Mg_2_Al-OH LDH followed by the intercalation with phosphate anion via the successive anion exchange reactions, ОН^−^ → Cl^−^ and Cl^−^ → H_2_PO_4_^−^, with high-power sonication. We demonstrate that the application of a kW-level ultrasound considerably accelerates all stages of the final product formation, namely hydration and both anion exchanges. Type of the intercalated phosphate anion and its arrangement in the interlayer have been concluded.

## Results and Discussion

**Figure 1:**
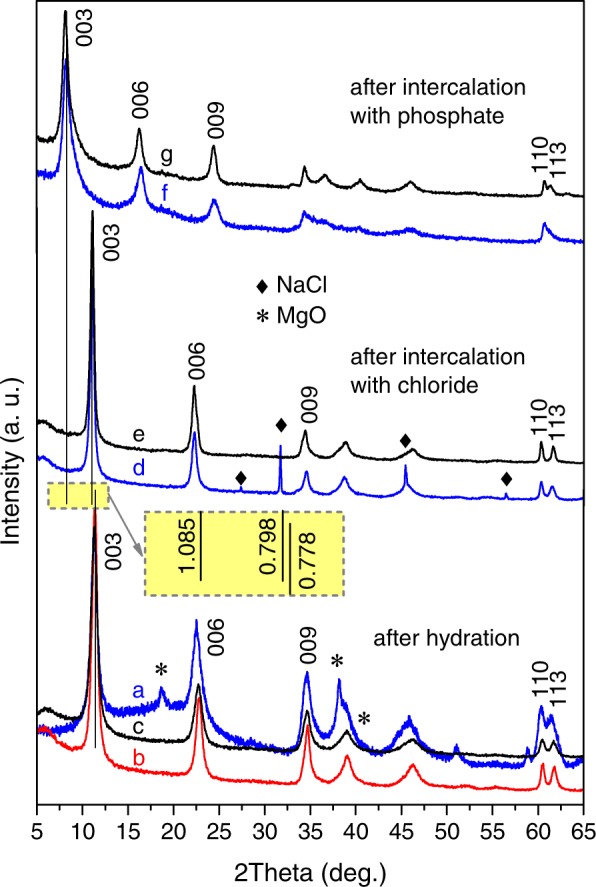
Typical XRD patterns of the products obtained after hydration of MMO resulted in formation of Mg_2_Al-OH LDH (**a**–**c**) and subsequent hydroxide-to-chloride (**e**,**d**) and chloride-to-phosphate (**f**,**g**) anion exchanges conducted in different conditions: (**a**) at room temperature for 24 h - Mg_2_Al-OH_(25°C/24h)_, (**b**) at 80 °C for 2 h - Mg_2_Al-OH_(80°C/2h)_ (**c**) with ultrasound applied for 30 min - Mg_2_Al-OH_(Sonic/30min)_, (**d**) at room temperature for 15 min - Mg_2_Al-Cl_(25°C/15min)_, (**e**) with ultrasound applied for 4 min - Mg_2_Al-Cl_(Sonic/4min)_, (**f**) at room temperature for 1 h - Mg_2_Al-H_*x*_PO_4(25°C/1h)_ and (**g**) with ultrasound applied for 8 min - Mg_2_Al-H_*x*_PO_4 (Sonic/8min)_. Inset: basal spacing values (in nm) of the respective LDH phases

It was found that formation of LDH phase by hydration of Mg_2_Al_(MMO)_ at room temperature is rather slow. Although some indications of the basal reflections corresponding to the LDH phase can be recognised already after first 15 min of the hydration, the characteristic LDH pattern is clearly seen only after 4 h. Traces of the MMO precursor disappear between 8 and 24 h. (For more details see Fig. S1 of Supplementary Information). However, even after 24 h, the diffraction reflections of the LDH phase are still wide that suggests a small average crystallite size and a broad size distribution (Fig. 1). Therefore, the reaction temperature was increased.

**Figure 2:**
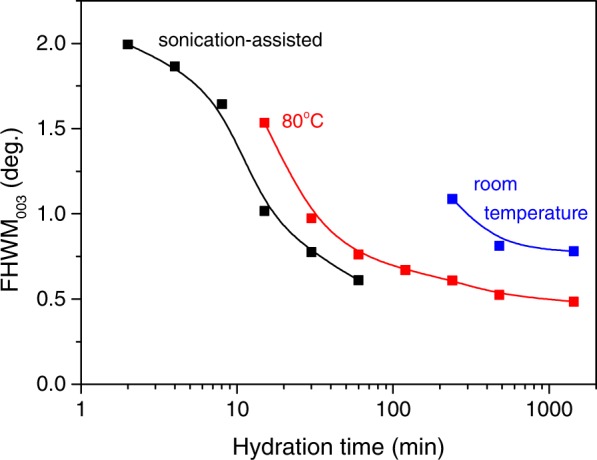
The full width at half maximum (FWHM) values of the 003 basal diffraction peaks of LDH phase obtained by hydration of Mg_2_Al MMO either at room temperature or at 80 °C or under applied high-power ultrasound as a function of hydration time. Notice the logarithmic time scale.

In the second set of experiments conducted at 80 °C, a single-phase Mg_2_Al-OH LDH was obtained already after 2-h hydration of Mg_2_Al_(MMO)_ (Fig. 1). It was observed that the diffraction peaks becomes narrower with further extension of the hydration process (Fig. S2 of Supplementary Information). Indeed, the values of full width at half maximum (FWHM) of the respected reflections were calculated to decrease monotonically as the hydration time is increased from 2 to 24 h (Fig. 2).

It follows from the comparison of the FWHM values of the basal reflections of the LDH phases crystallized either at room temperature or at 80 °C that the reaction rate increased by more than factor of 20.

In the third set of experiments, high-power ultrasound was applied to prepare LDHs of the same composition. It was found that the sonication assisted reaction occurs faster in comparison with that performed at 80 °C under vigorous mechanical stirring (cf.: Figs S2 and S3). A single phase Mg_2_A-OH LDH was obtained after 30 min of ultrasound treatment (Fig. 1). Moreover, in case of the sonication assisted reaction, the LDH as the main phase appeared already after 2-min treatment; however, the hydration was still uncomplete: traces of the MMO precursor were present in the product prepared for 15 min.

The Mg_2_Al-OH_(25°C/24h)_ LDH was chosen as a starting material to study anion-exchange processes. A hydroxide-to-phosphate direct anion exchange was unsuccessful. A 24-h immersion of Mg_2_Al-OH LDH in a 0.1M Na_2_HPO_4_ solution resulted in no visible change of the peak positions in XRD pattern regardless of increase of the solution temperature to 80°C or application of high-power ultrasound. Therefore, a two-step hydroxide → chloride → phosphate process was attempted. Due to a small difference size of OH^−^ and Cl^−^, the shift in the basal diffraction reflections to lower 2theta angles was also rather small (Fig. 1). The Cl^−^-intercalation reaction at room temperature was found to take 15 min, while the sonication assisted anion exchange was completed in 4 min. (For more details see Figs S4 and S5 of Supplementary Information).

The Mg_2_Al-Cl_(25°C/24h)_ LDH was used as starting material to intercalate phosphate anions. The chloride-to-phosphate anion exchange was manifested in the shift of the basal reflections in the XRD patterns towards lower 2theta angles indicating a considerable increase of the interlayer distance. The anion exchange took about 30 min in the case of standard mixing procedure at room temperature, while the exchange was complete in 4 min when the high-power ultrasound was applied. The broader reflections were observed in the XRD patterns of Mg_2_Al-H_*x*_PO_4_ LDHs (Fig. 1) in comparison with those seen in the patterns of the hydroxide-intercalated and chloride-intercalated LDHs. This can indicate some disorder in arrangement of the phosphate anions into the interlayer. It has also been revealed that the interlayer distance is not fixed after a 30-min exchange (Fig. S6 of Supplementary Information). Moreover, the interlayer distance is a non-monotonic function of the exchange reaction time that may suggest two or more competing arrangements of phosphate anions in the interlayer. It should be stressed here that in case of high-power sonication assisted reaction no such variation in the interlayer distance with time has been observed (Fig. S7 of Supplementary Information).

The lattice parameters *a* and *c* of all the obtained layered double hydroxides were calculated using the angle positions of the diffraction peaks (003), (006) and (110) as *c* = 3/2[*d*_(003)_ + 2*d*_(006)_] and *a* = 2*d*_(110)_^1^. The parameter *a* reflects the average distance between cations in the double metal hydroxide layer, while the parameter *c* relates to the basal spacing (*d*) as *c* = 3*d*. The results of calculations are listed in Table 1. The maximum absolute errors in determination of the parameters *c* and *a* were 0.15 Å and 0.01 Å, respectively. The *a*-parameter values of the obtained Mg_2_Al LDHs intercalated with either hydroxide, chloride or phosphate are equal within the experimental error. The difference between the *c*-parameter values of the respective Mg_2_Al-OH and Mg_2_Al-Cl LDHs is in good agreement with the previously reported data^3^ and the references therein (about 0.4–0.6 Å). As already mentioned before, the observed variation in the values of parameter *c* of the LDHs intercalated with phosphate (Table 1) may imply several possibilities of arrangement of phosphate anion.

**Table 1:** The interplanar distances used for calculation and the calculated lattice parameters (a,c) of the LDHs obtained via hydration and anion exchanges.

Sample ID	*d*_(003)_, Å	*d*_(006)_, Å	*d*_(110)_, Å	*c*, Å	*a*, Å
Mg_2_Al-OH_(80°C/2h)_	7.843	3.906	1.532	23.483	3.064
Mg_2_Al-OH_(Sonic/30min)_	7.908	3.930	1.536	23.652	3.070
Mg_2_Al-Cl_(25°C/15min)_	7.986	3.985	1.534	23.934	3.068
Mg_2_Al-Cl_(Sonic/4min)_	8.004	3.993	1.527	23.985	3.054
Mg_2_Al-H_*x*_PO_4 (25°C/1h)_	9.992	5.277	1.523	30.819	3.049
Mg_2_Al-H_*x*_PO_4 (Sonic/8min)_	10.779	5.380	1.525	32.309	3.050

**Figure 3:**
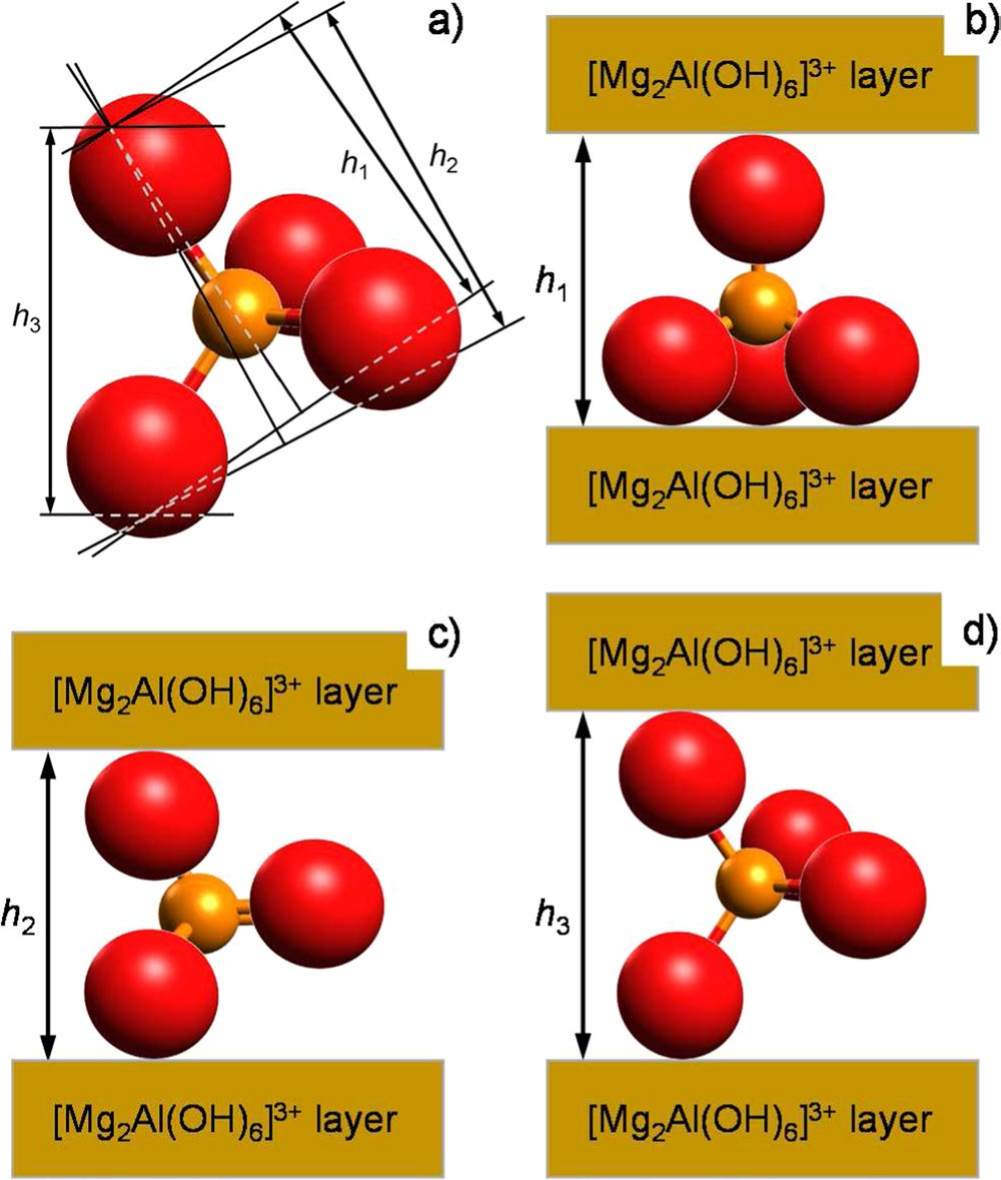
Schematic representations of (**a**) the most characteristic dimensions of H_*x*_PO_4_^(3−*x*)−^ anion and the respective orientations of the anion in the Mg_2_Al LDH interlayer: (**b**) the space height of the tetrahedron is perpendicular to the hydroxide layer, (**c**) the height of triangular face of the tetrahedron is perpendicular to the hydroxide layer, and (**d**) edge length of the tetrahedron is perpendicular to the hydroxide layer. Hydrogen ions are not shown.

Using the Table 1 data and taking into account the thickness of the Mg-Al hydroxide layer (*d*_0_ = 4.77 Å^38^), the calculated interlayer gallery height is 6.00 Å and 5.30 Å for Mg_2_Al-H_*x*_PO_4(Sonic/30min)_ and Mg_2_Al-H_*x*_PO_4(25°C/1h)_ LDHs, respectively. The chloride-to-phosphate exchange was conducted at pH 7.5 (see Experimental). At such pH, the most likely anions in the solution are H_2_PO_4_^−^ and HPO_4_^2−^^13^. These anions are almost identical in shape and size; the only difference is the number of protons. To model the arrangement of phosphate anion one can use an approach similar to that reported in ref.^39^ for the pyrovanadate-intercalated Zn_2_Al LDH. Provided that a PO_4_ tetrahedron is regular, the anion has the following characteristic dimensions: along the space height - ${h}_{1}=2R({{\rm{O}}}^{2-})+\sqrt{\frac{2}{3}}{a}_{0}$, along the height of triangular face - ${h}_{2}=2R({{\rm{O}}}^{2-})+\frac{\sqrt{3}}{2}{a}_{0}$, and along the edge length - ${h}_{3}=2R({{\rm{O}}}^{2-})+{a}_{0}$ (Fig. 3a), where ${a}_{0}=\frac{4}{\sqrt{6}}[R({{\rm{O}}}^{2-})+R({{\rm{P}}}^{5+})]$; *R*(O^2−^) and *R*(P^5+^) are radii of the constituting ions: 1.36 Å and 0.35 Å, respectively. The calculated values are *h*_1_ = 5.00 Å, *h*_2_ = 5.14 Å, and *h*_3_ = 5.51 Å.

One can suggest from a comparison of these values with the interlayer heights observed in the Mg_2_Al-H_*x*_PO_4_ LDHs ($h=\tfrac{c}{3}-{d}_{0}$, Table 1) that the most probable orientation of the phosphate anion is the following: a tetrahedron edge is perpendicular to the hydroxide layer (Fig. 3d).

**Figure 4:**
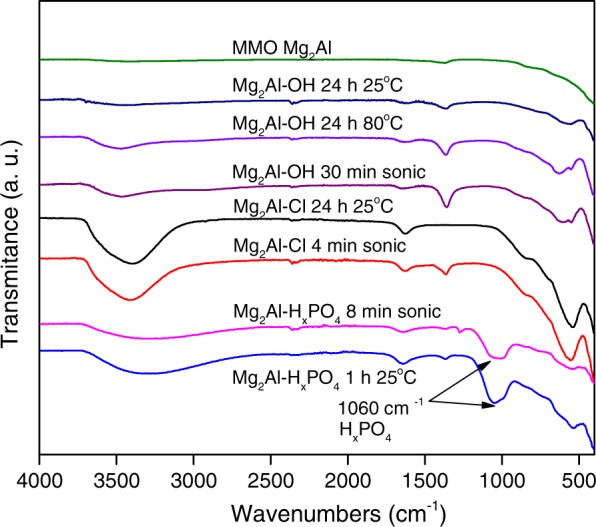
FTIR spectra of LDHs obtained via hydration and anion exchanges at the conditions indicated in the plot labels.

The FTIR spectra of the samples before and after completion of hydration or anion-exchange processes are shown in Fig. 4. The broad absorption bands observed at around 3600–3000 cm^−1^ and weaker bands at 1640–1650 cm^−1^ could be attributed to the stretching vibrations of -OH groups from the hydroxide layers and from the intercalated water molecules. Very weak absorption bands in the range of 1360–1370 cm^−1^ can be attributed to the asymmetric vibrations modes of CO_3_^2−^. Although decarbonized water was used, the experiments on hydration and anion-exchanges were in open air; therefore, some contamination of samples with carbonate was possible. In the FTIR spectra of the phosphate-intercalated LDHs, the intense bands situated at ~1060 cm^−1^ which could be assigned to vibrations in phosphate tetrahedron^14^ are clearly seen.

The obtained LDHs were analysed using ICP-OES. The results of analytical determination of elements in the samples are presented in Table 2. Evidently, the stoichiometry of magnesium and aluminium in the sol-gel derived Mg_2_Al LDHs is close to the nominal one regardless of the methods used for acceleration of the hydration and the anion-exchanges. The Mg/Al/P ratio in the phosphate-intercalated LDHs is close to 2/1/1. Taking into account the generic formula of the M^II^-M^III^ LDH (see *Introduction*), the obtained ratio indicates the type of the intercalated phosphate anion, namely dihydrogen phosphate, H_2_PO_4_^−^. The chemical composition of the phosphate-intercalated LDH produced in this work can be represented as Mg_0.67_Al_0.33_(OH)_2_(H_2_PO_4_)_0.33_·*z*H_2_O.

**Table 2:** Results of the ICP-OES elemental analysis of the obtained LDHs.

Sample ID	*n*(Mg)	*n*(Al)	*n*(P)	Mg/Al ratio	Mg/Al/P ratio
Mg_2_Al-OH_(25°C/24 h)_	0.235	0.119	—	1.975/1	—
Mg_2_Al-OH_(Sonic/30 min)_	0.279	0.140	—	1.993/1	—
Mg_2_Al-Cl_(25°C/15min)_	0.412	0.189	—	2.180/1	—
Mg_2_Al-Cl_(Sonic/4min)_	0.428	0.201	—	2.123/1	—
Mg_2_Al-H_2_PO_4(Sonic/8min)_	0.267	0.126	0.142	—	2.119/1/1.127

**Figure 5:**
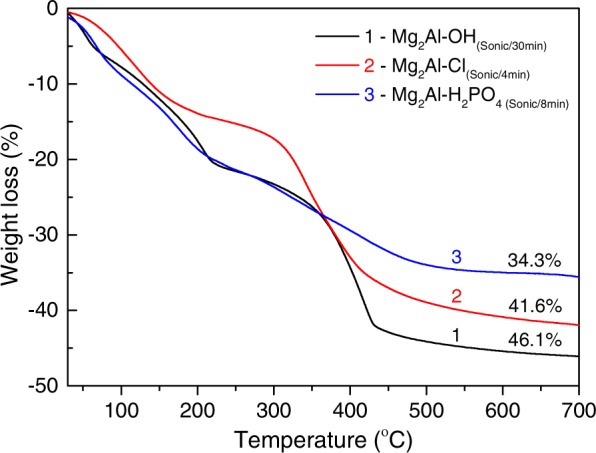
TG analysis curves of the LDHs obtained via sonication-assisted hydration and anion exchanges.

TG analysis curves of the Mg_2_Al-OH, Mg_2_Al-Cl and Mg_2_Al-H_2_PO_4_ prepared with application of high-power ultrasound are shown in Fig. 5. The curves of the respective LDHs prepared without sonication were found to be very similar (cf.: Fig. S8 in Supplementary Information).

The obtained weight loss values were used to calculate the relative amount (per formula unit) of crystal water (*z*) in the Mg_2_Al-H_2_PO_4_ LDH. It was assumed that the final product of all these LDHs after TG heating to 700°C is the same, namely MMO with the Mg/Al ratio = 2. The value of *z* was calculated to be close to 0.7. It means that the phosphate-intercalated LDH contains one H_2_PO_4_^−^ anion and two water molecules per three formula units. Taking into account the hexagonal symmetry of the LDH structure, the available interlayer volume for three formula units can be found as ${V}_{available}=3(\frac{c}{3}-{d}_{0})\frac{\sqrt{3}}{2}{a}^{2}$^10^. The total volume of one phosphate anion (whose volume is mainly volume of four oxygen atoms) and two H_2_O molecules (two oxygen atoms) is roughly ${V}_{species}=6{(2R({{\rm{O}}}^{2-}))}^{3}$. The calculation of the available volume using the smallest observed *c*-parameter value in a phosphate-intercalated LDH (Table 1) gives *V*_*available*_ ~129 Å^3^, while the maximum total volume of the intercalated species *V*_*species*_ ~120 Å^3^, which are in very good agreement.

**Figure 6:**
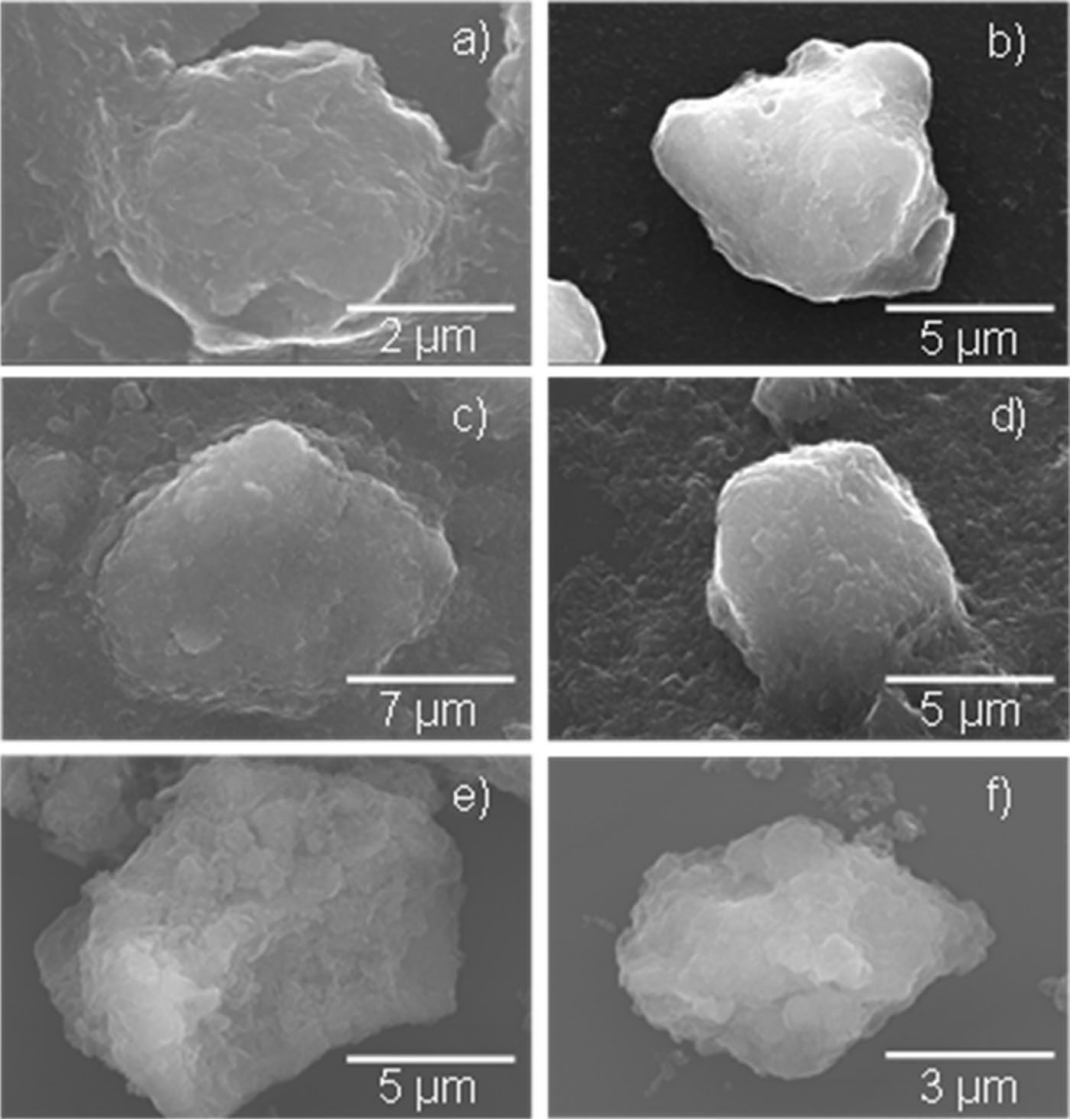
SEM micrographs of the LDH powders prepared via hydration of MMO followed by anion exchanges at different temperatures without and with application of high-power ultrasound: (**a**) Mg_2_Al-OH_(80°C/24h)_, (**b**) Mg_2_Al-OH_(Sonic/30min)_, (**c**) Mg_2_Al-Cl_(25°C/15min)_, (**d**) Mg_2_Al-Cl_(Sonic/4min)_, (**e**) Mg_2_Al-H_2_PO_4 (25°C/1h)_ and (**f**) Mg_2_Al-H_2_PO_4(Sonic/8min)_.

The morphology of the prepared LDH samples was investigated by SEM and STEM. The SEM micrographs are shown in Fig. 6. The particles agglomerated of flake-like crystallites were observed in all samples.

**Figure 7:**
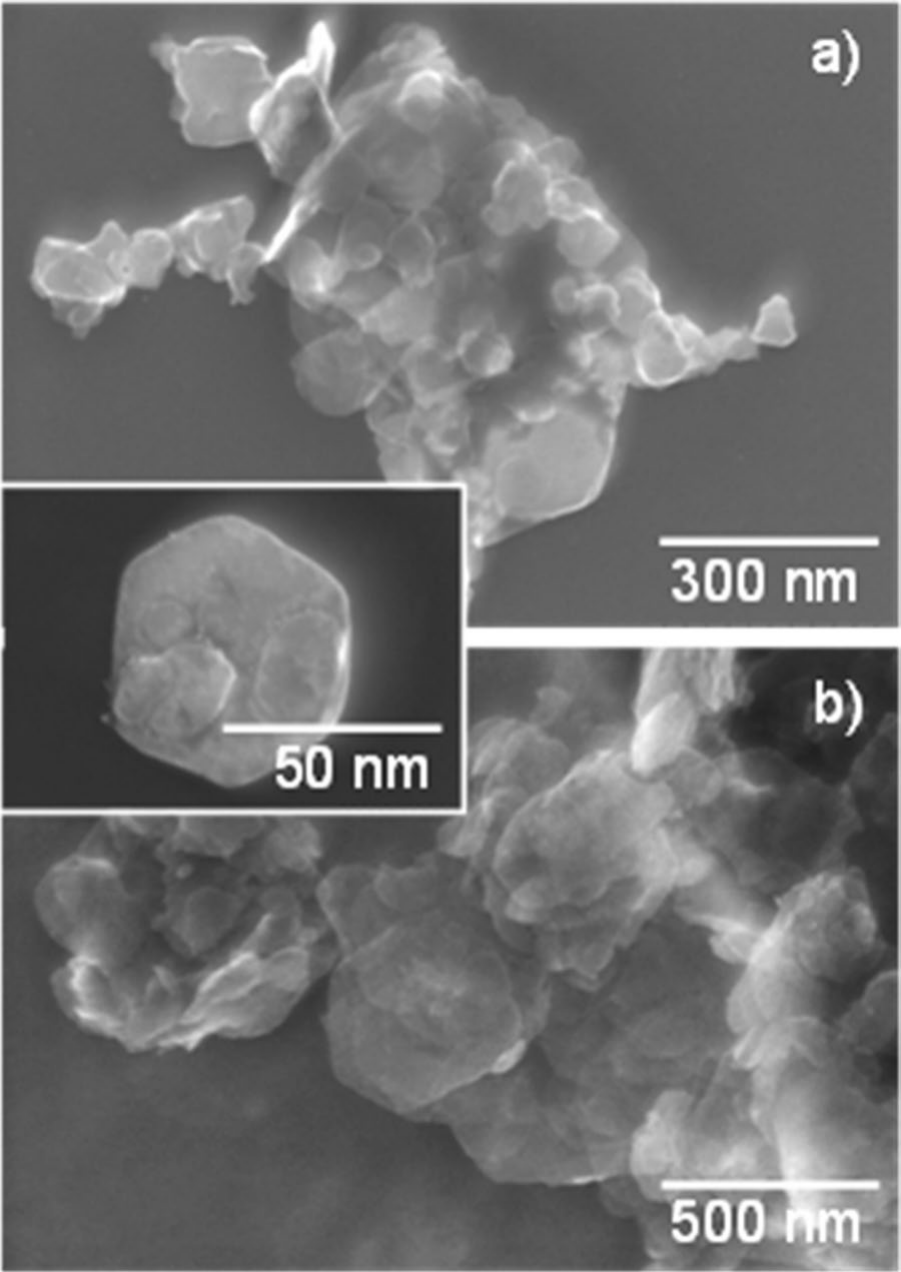
STEM micrographs of the LDHs obtained via sonication-assisted hydration and anion exchanges: (**a**) Mg_2_Al-Cl_(Sonic/4min)_ and (**b**) Mg_2_Al-H_2_PO_4(Sonic/8min)_. Inset shows a detached crystallite of Mg_2_Al-OH_(Sonic/30min)_ LDH.

The STEM micrographs (Fig. 7) reveal characteristic hexagonal shape of the flake-like LDH crystallites. It should be stressed here that no effect of the preparation conditions (increase of temperature, application of ultrasound) on size and shape of LDH particles nor crystallites has been observed.

## Conclusions

Stoichiometric magnesium-aluminium layered double hydroxide with the Mg/Al ratio = 2 and intercalated with hydroxide anion (Mg_2_Al-OH LDH) is prepared by hydration of mixed metal Mg_2_Al oxide (MMO) obtained using the aqueous sol-gel based method. The complete transformation of the MMO into Mg_2_Al-OH takes about 24 h at room temperature and 2 h at 80 °C. When high-power ultrasound is applied, the transformation is complete in 30 min.

Deintercalation of OH^−^ from Mg_2_Al-OH resulted in the formation of Mg_2_Al-Cl LDH is done via anion exchange in a HCl/NaCl solution. The Cl^−^-intercalation reaction at room temperature takes 15 min, while the sonication assisted anion exchange completes in 4 min.

Mg_2_Al-H_*x*_PO_4_ LDH is prepared from Mg_2_Al-Cl LDH via anion exchange in a Na_2_HPO_4_ solution at pH 7.5. The exchange takes about 30 min at room temperature and 4 min when high-power ultrasound is applied. The intercalated phosphate anion is dihydrogen phosphate, H_2_PO_4_^−^.

The used procedure of the formation of LDH intercalated with hydroxide via hydration of sol-gel prepared MMO followed by the deintercalation of OH^−^ and the intercalation with phosphate via a two-step anion exchange appear to be a promising way for production of stoichiometric LDH intercalated with functional species. High-power sonication considerably accelerates the formation of LDH phase from mixed metal oxides and the successive anion exchange processes.

## Experimental Methods

### Materials

The following materials were used for synthesis and anion modification of LDHs: Al(NO_3_)_3_·9H_2_O (98.5%, Chempur), Mg(NO_3_)_2_·6H_2_O (99%, Chempur), NaCl (99.9% Chempur), NaH_2_PO_4_·2H_2_O (>98% Carl Roth), Na_2_HPO_4_·2H_2_O (>98% Scharlau), HNO_3_ (66%, REACHEM s.r.o.), C_2_H_6_O_2_ (99%, Sigma-Aldrich), HCl (36%, Chempur). Deionised and decarbonised water was used for syntheses, preparation of solutions and washing of final products.

### Synthesis of Mg_2_Al-hydroxide LDH via aqueous sol-gel method

Mg_2_Al precursor was prepared by mixing the solutions of the appropriate metal nitrates with a molar ratio of Mg/Al = 2:1. The nitrates were dissolved in 50 ml of distilled water with addition of 50 ml of a 0.2 M nitric acid. The solution was stirred for 1 h at 80 °C. Then 2 ml of ethylene glycol were added under continuous stirring for 4 h at the same temperature. After slow evaporation of solvent, the obtained gel was dried at 120–140 °C for 10–14 h. The Mg_2_Al MMO was obtained by calcination of the precursor gel at 650 °C for 3 h (hereafter labelled as Mg_2_Al_(MMO)_). This calcination temperature was shown to be optimal for production of Mg-Al MMO that demonstrate the most complete transformation into a respective LDH phase upon hydration^5^. Hydration of the Mg_2_Al_(MMO)_ powder in water was carried out under three different conditions, namely i) at room temperature with a vigorous mechanical stirring for defined time X of 15, 30 min, 1, 2, 4, 8, and 24 h; ii) at 80 °C with stirring for 15, 30 min, 1, 2, 4, 8, and 24 h; and iii) at high-power sonication applied for 2, 4, 8, 15, and 30 min. The final powder samples were obtained by vacuum filtration and drying at 60 °C for 30 min (Mg_2_Al-OH_(25°C/X)_, Mg_2_Al-OH_(80°C/X)_ and Mg_2_Al-OH_(Sonic/X)_, respectively).

### Anion exchange in *Mg*_*2*_*Al LDH* from hydroxide to chloride

Anion exchange was performed in 250 ml of a 1 M NaCl solution with addition of 0.3 ml of hydrochloric acid (36%). 1 g of Mg_2_Al-OH_(80°C/24h)_ was used. The reaction was carried out under two different conditions: i) at 25 °C with stirring for 15, 30 min, 1, 2, 4, 8, and 24 h; and ii) at high-power sonication applied for 2, 4, 8, 15, and 30 min. The final products (Mg_2_Al-Cl_(25°C/X)_ and Mg_2_Al-Cl_(Sonic/X)_, respectively) were obtained by vacuum filtration without any additional washing followed by drying for 30 min at 60 °C.

### Anion exchange in *Mg*_*2*_*Al LDH* from chloride to phosphate

1 g of Mg_2_Al-Cl_(25°C/24h)_ was immersed into a 0.1 M Na_2_HPO_4_ solution with addition of small amount of NaH_2_PO_4_ to adjust the pH value to 7.5. This reaction was carried out either at 25 °C with vigorous mechanical stirring or at high-power sonication applied for the same respective time intervals as those used the hydroxide-to-chloride exchange (see part *Anion exchange in* Mg_2_Al LDH *from hydroxide to chloride*). The samples labelled as Mg_2_Al-H_*x*_PO_4 (25°C/X)_ and Mg_2_Al-H_*x*_PO_4 (Sonic/X)_, respectively, were obtained by vacuum filtration without any additional washing followed by drying for 30 min at 60 °C.

### Characterization techniques

The phase content and the crystal structure of the obtained samples were characterized using a PANalytical X’Pert Powder diffractometer (Ni-filtered Cu Kα radiation, step 0.02^o^, exposition time ~1.5 s per step) over the range of 5–70° at room temperature. The morphology of the samples was investigated by scanning transmission electron microscope (STEM) Hitachi HD-2700 operated at 200 kV and by scanning electron microscope (SEM) Hitachi S4100, 30 kV. Thermogravimetric (TG) analysis was carried out using a Perkin Elmer STA6000 apparatus. TG data were collected upon heating the samples from 30 to 700 °C at a rate of 10 °C/min. Mg, Al, and P elemental analysis was performed by inductive coupled plasma optical emission spectroscopy (ICP-OES) using a Perkin Elmer 3300 instrument. Fourier transform infrared (FTIR) spectra of the samples were recorded with a Perkin Elmer spectrum BX FTIR spectrometer, averaging 100 scans with a nominal resolution of 4 cm^−1^. Sonication-assisted experiments were performed using a VCX 1500 Sonics processor (max output power 1.5 kW at 20 kHz) equipped with a continuous flow cell.

## Supplementary information

Supplementary_Information
