# Supplementary material for: Sonication accelerated formation of Mg-Al-phosphate layered double hydroxide via sol-gel prepared mixed metal oxides
Source: Sci Rep. 2019 Jul 18;9:10419. doi: 10.1038/s41598-019-46910-5 (PMC6639339; doi:10.1038/s41598-019-46910-5)
Supplement: Supplementary file 1 — Supplementary_Information [file 41598_2019_46910_MOESM1_ESM.pdf]

Sonication accelerated formation of Mg-Al-phosphate layered double hydroxide via sol-gel prepared mixed metal oxides

Denis Sokol[1], Daniel E.L. Vieira[2], Aleksej Zarkov[1], Mário G. S. Ferreira[2], Aldona Beganskiene[1], Vasili V. Rubanik[3], Aleksandr D. Shilin[3], Aivaras Kareiva[1,#], and Andrei N. Salak[2,*]

[1]*Institute of Chemistry, Faculty of Chemistry and Geosciences, Vilnius University, Naugarduko 24, LT-03225 Vilnius, Lithuania*
[2]*Department of Materials and Ceramics Engineering and CICECO – Aveiro Institute of Materials, University of Aveiro, 3810-193 Aveiro, Portugal*
[3]*Institute of Technical Acoustics of National Academy of Sciences Belarus, Lyudnikov Avenue, 13, 210009 Vitebsk, Belarus*

# aivaras.kareiva@chf.vu.lt
* salak@ua.pt – corresponding author

Supplementary Information

The samples nomenclature

- $Mg_2Al_{(MMO)}$ – mixed metal oxide with the cation ratio Mg/Al=2 obtained by calcination of nanopowder prepared using sol-gel based method.
- $Mg_2Al\text{-}OH_{(T°C/X)}$, $Mg_2Al\text{-}OH_{(Sonic/X)}$ – hydroxide-intercalated Mg_2Al LDHs produced by hydration of $Mg_2Al_{(MMO)}$ for time X either at fixed temperature T(°C) or at a high-power sonication applied, respectively.
- $Mg_2Al\text{-}Cl_{(T°C/X)}$, $Mg_2Al\text{-}Cl_{(Sonic/X)}$ – chloride intercalated Mg_2Al LDHs prepared by anion exchange from $Mg_2Al\text{-}OH_{(25°C/24h)}$ for time X either at fixed temperature T(°C) or at a high-power sonication applied, respectively.
- $Mg_2Al\text{-}H_xPO_{4(T°C/X)}$, $Mg_2Al\text{-}H_xPO_{4(Sonic/X)}$ – phosphate intercalated Mg_2Al LDHs prepared by anion exchange from $Mg_2Al\text{-}Cl_{(25°C/24h)}$ for time X either at fixed temperature T(°C) or at a high-power sonication applied, respectively.

X-ray diffraction (XRD) study

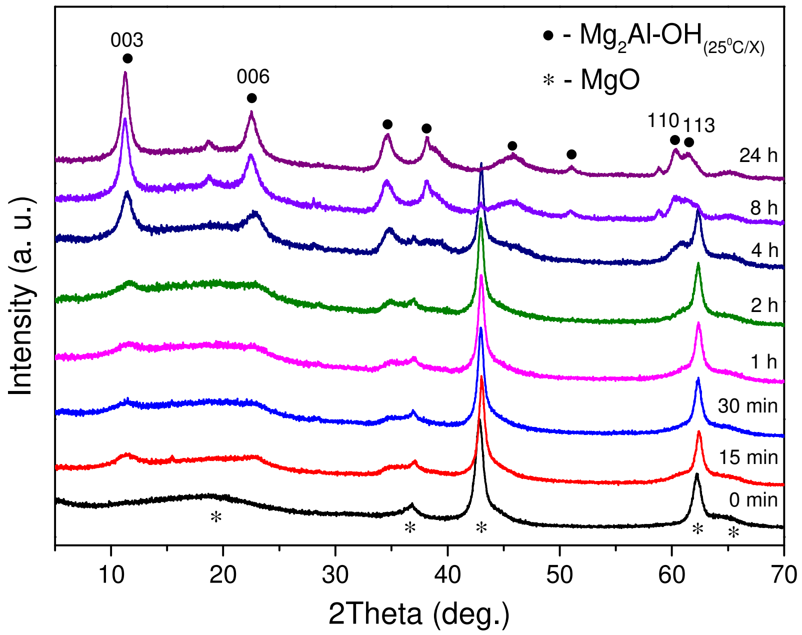

Figure S1. XRD patterns of the products obtained as a result of hydration of Mg_2Al MMO at room temperature for different time from 15 min to 24 h. The pattern of the as-prepared $Mg_2Al_{(MMO)}$ is shown for a comparison.

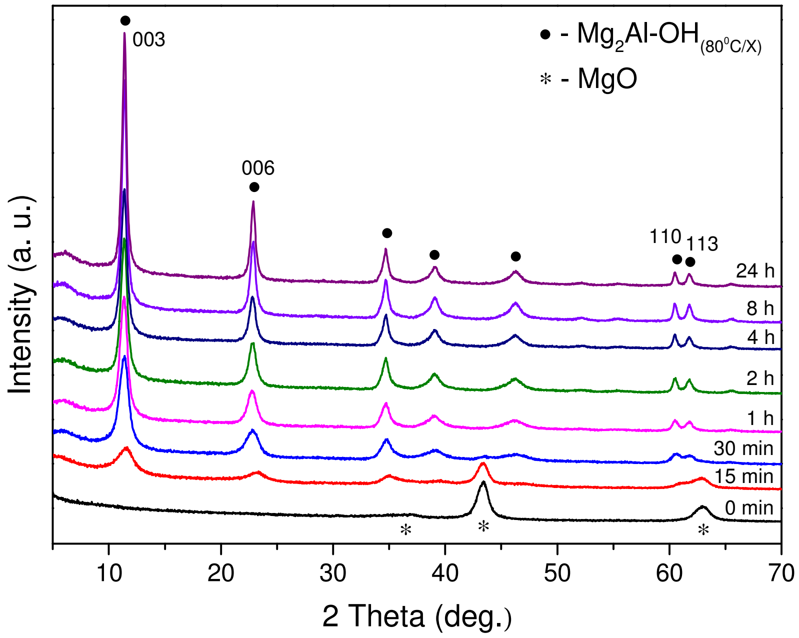

Figure S2. XRD patterns of the products obtained as a result of hydration of Mg_2Al MMO at 80^0C for different time from 15 min to 24 h. The pattern of the as-prepared $Mg_2Al_{(MMO)}$ is shown for a comparison.

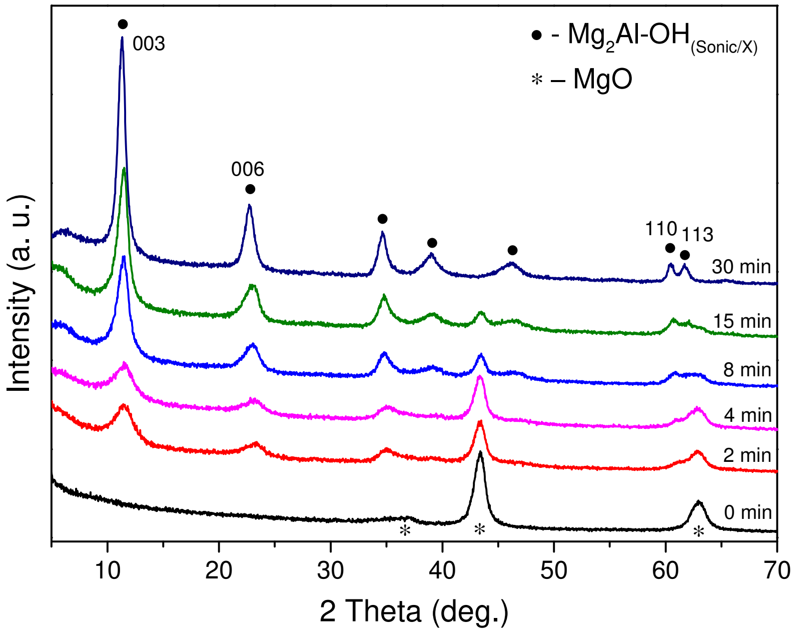

Figure S3. XRD patterns of the products obtained as a result of high-power sonication accelerated hydration of Mg_2Al MMO for different time from 2 to 30 min. The pattern of the as-prepared $Mg_2Al_{(MMO)}$ is shown for a comparison.

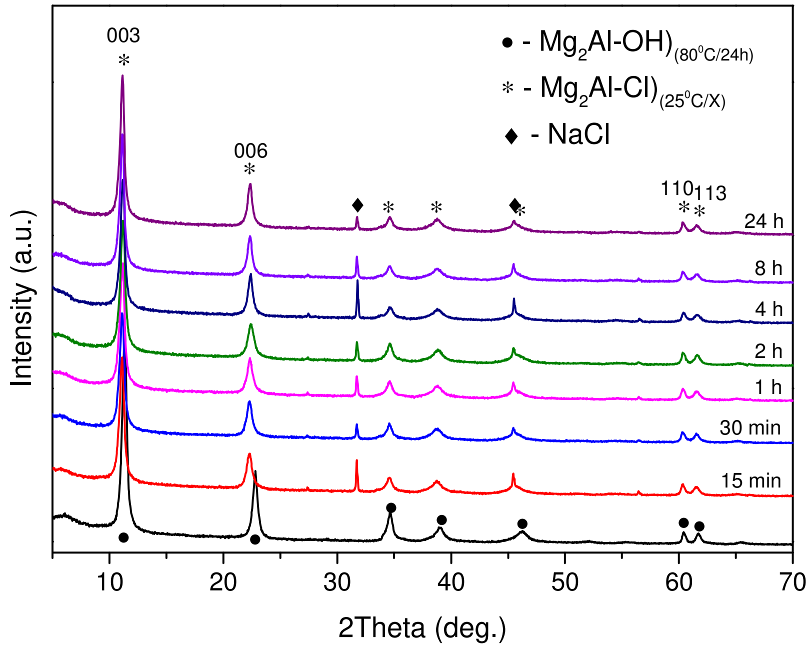

Figure S4. XRD patterns of the products obtained as a result of a hydroxide-to-chloride anion exchange in $Mg_2Al\text{-}OH_{(80°C/24h)}$ LDH at room temperature for different time from 15 min to 24 h.

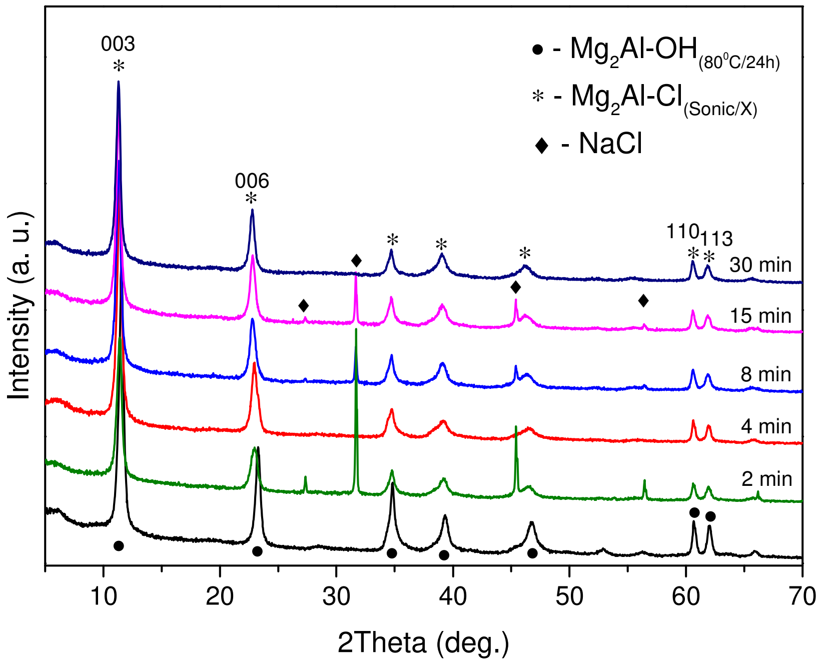

Figure S5. XRD patterns of the products obtained as a result of a high-power sonication accelerated hydroxide-to-chloride anion exchange in $Mg_2Al\text{-}OH_{(80°C/24h)}$ LDH for different time from 2 to 30 min.

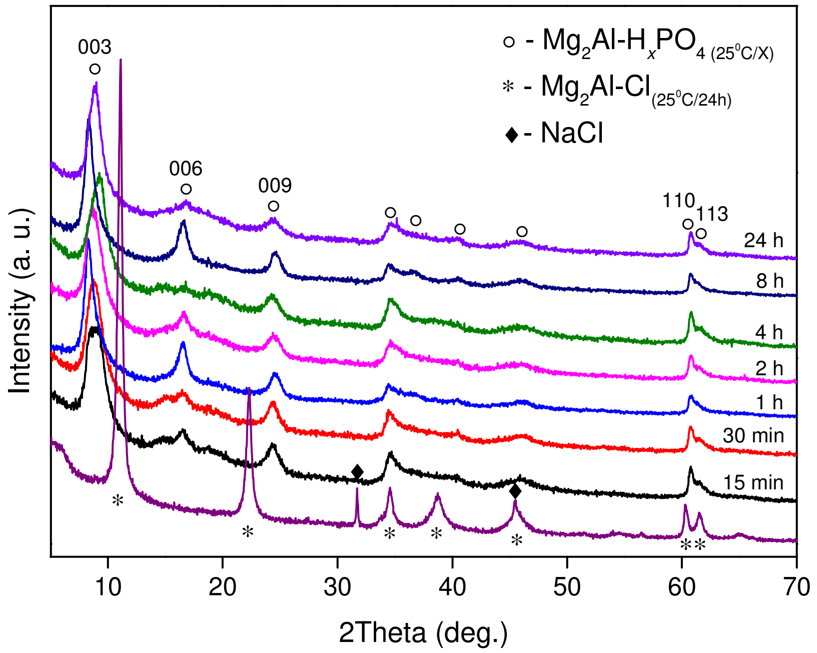

Figure S6. XRD patterns of the products obtained as a result of a chloride-to-phosphate anion exchange in $Mg_2Al\text{-}Cl_{(25°C/24h)}$ LDH at room temperature for different time from 15 min to 24 h.

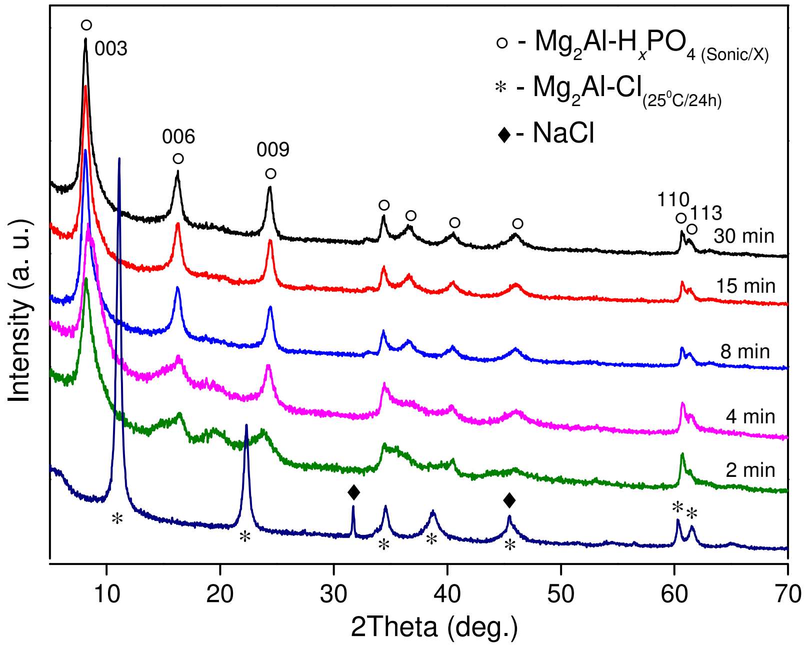

Figure S7. XRD patterns of the products obtained as a result of a high-power sonication accelerated chloride-to-phosphate anion exchange in $Mg_2Al\text{-}Cl_{(25°C/24h)}$ LDH for different time from 2 to 30 min.

Thermogravimetric (TG) analysis

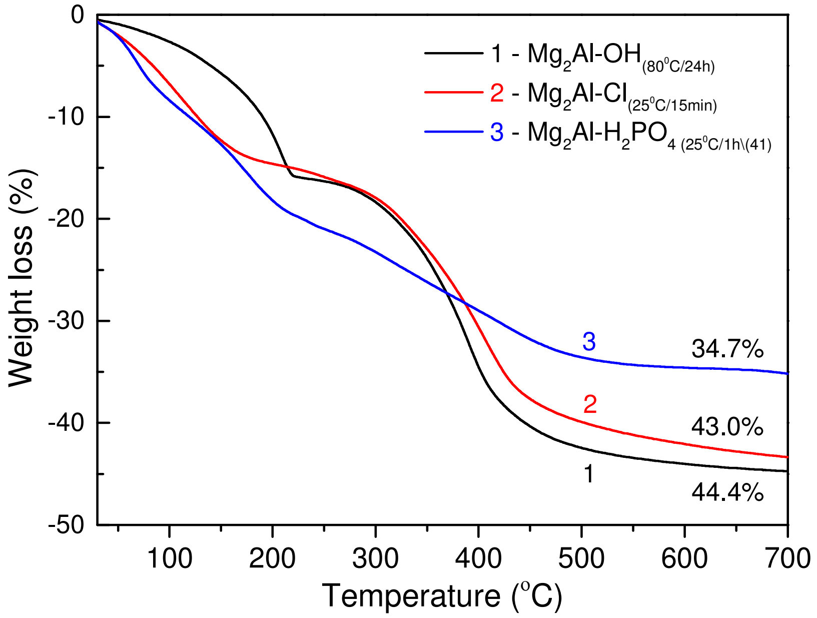

Figure S8. TG analysis curves of the LDHs obtained via hydration and anion exchanges without application of high-power ultrasound.
